# Rapid and reliable detection of *Leishmania* antibodies in canine serum with double-antigen sandwich homogeneous chemical luminescence

**DOI:** 10.1186/s13071-024-06389-0

**Published:** 2024-07-30

**Authors:** Xiangjun Zhao, Licai Ma, Yipeng Jin, Herman W. Barkema, John P. Kastelic, Lu Wang, Kai Wen, Gang Liu

**Affiliations:** 1 National Key Laboratory of Veterinary Public Health Safety, Beijing Key Laboratory of Detection Technology for Animal Derived Food Safety, Beijing Laboratory for Food Quality and Safety, College of Veterinary Medicine, China Agricultural University, Beijing, 100193 People’s Republic of China; 2 Beijing Weideweikang Biotechnology Co., Ltd, Beijing, 100080 People’s Republic of China; 3 Faculty of Veterinary Medicine, University of Calgary, Calgary, AB Canada; 4 Veterinary Teaching Hospital, College of Veterinary Medicine, China Agricultural University, Beijing, China

**Keywords:** Leishmaniasis, Serological testing, Homogeneous chemiluminescent analysis

## Abstract

**Background:**

Leishmaniasis, caused by *Leishmania* spp. parasites, is an important zoonotic disease globally, posing severe threats to humans and animals. In the absence of effective vaccines, reliable serological diagnostic methods are critical for disease control. However, the enzyme-linked immunosorbent assay (ELISA) and immunochromatographic assay have limitations due to complexity, time required and/or sensitivity. Therefore, our objective was to develop an accurate, rapid and user-friendly detection method of canine leishmania antibody based on double-antigen sandwich homogeneous chemical luminescence.

**Methods:**

Homogeneous chemiluminescent technology was employed, and expressed recombinant fusion proteins containing full-length K9, K39 and K26 repeat sequences were used as diagnostic antigens. To establish a dual-antigen sandwich serological assay capable of detecting various antibody types, a factorial design was used to optimize concentrations of diagnostic antigen-receptor microspheres and of biotinylated diagnostic antigens, as well as of reaction solution composition and reaction duration. To evaluate and validate this newly developed method, we collected 41 *Leishmania*-positive serum samples, 30 *Leishmania*-negative control serum samples and 78 clinical serum samples for which no diagnostic information was available. Comparative analyses were performed using parasitological testing and an indirect ELISA as reference methods, focusing on diagnostic sensitivity and specificity.

**Results:**

Sodium dodecyl sulfate–polyacrylamide gel electrophoresis confirmed the purification of the diagnostic antigens, which exhibited clear bands without impurities. Based on results from the 41 *Leishmania*-positive samples and 30 *Leishmania*-negative samples, there was sufficient sensitivity to detect samples diluted up to 256-fold, with analytical specificity of 100%. Overall diagnostic sensitivity was 100% and diagnostic specificity was 93.3%. Diagnostic performance was highly consistent between the newly developed method and the indirect ELISA (Kappa = 0.82, *P* < 0.01). Testing could be completed within 35 min with the new method

**Conclusions:**

We have developed a novel double-antigen sandwich homogeneous chemical luminescence method to detect canine *Leishmania* antibodies, with high sensitively and specificity, a short incubation interval and a simple protocol. This streamlined approach not only offers a sensitive and efficient method for clinical diagnosis but also has great potential for use in automated testing.

**Graphical Abstract:**

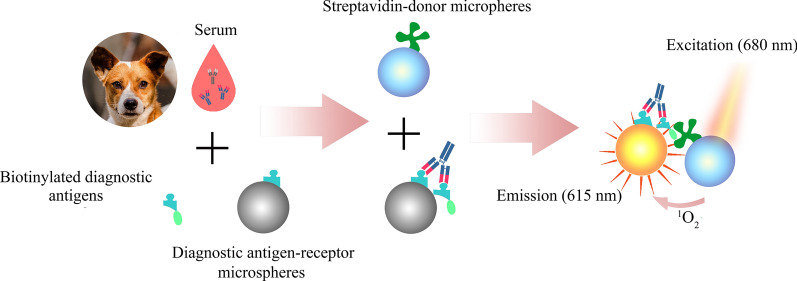

## Background

Leishmaniasis is a zoonotic disease caused by infection with *Leishmania* parasites that are transmitted by insect vectors. It can be categorized into three main forms depending on the clinical presentation: visceral leishmaniasis (VL), cutaneous leishmaniasis (CL) and mucocutaneous leishmaniasis (ML) [1]. Without timely treatment, the disease can have a high fatality rate [2]. In the absence of an effective vaccine, timely and accurate detection is a primary focus in disease control [3], with antibody testing widely applied. The diagnostic efficacy of serological testing is better for VL compared to CL and ML [3].

Before the founding of the People’s Republic of China, leishmaniasis used to be prevalent in the region north of the Yangtze River, over an area involving 16 provinces and autonomous regions. Through implementation of a concerted nationwide control program, the disease has been eliminated in major epidemic areas [4], but due to poor healthcare conditions, the disease has persisted as an endemic issue in the northwest region of China. However, the re-emergence of mountain-type zoonotic VL (MT-ZVL) has been reported recently in the central area of China, including the Yanshan-Taihangshan mountain areas (Henan, Hebei and Beijing) [5]. Notably, the incidence of MT-ZVL has rocketed rapidly in the last 5 years, accounting for 94.4% of the total number of VL cases in China [6]. In 2023, our group reported the re-emergence of canine leishmaniasis in Beijing, with a prevalence of 4.7% in dogs living in mountain areas of Beijing [7]. Taken together, these studies indicate that leishmaniasis remains a serious public health problem in China.

Clinical diagnosis of canine leishmaniasis (CanL) remains challenging due to a wide range of clinical signs, and many infected dogs may remain asymptomatic [8]. Therefore, it is necessary to develop accurate diagnostic methods for the rapid clinical diagnosis of CanL. Parasitological examination of bone marrow, lymph nodes and other tissue aspirates is considered to be the gold standard for diagnosing leishmaniasis due to high specificity. However, this method has been found to have a lower sensitivity, ranging from 52% to 85% in bone marrow aspirates, despite a higher sensitivity (93.1–98.7%) in splenic aspirates [9]. Direct observation of parasites in tissue aspirates is the gold standard for VL diagnosis, but it is only performed by a few medical centers due to its complexity and need for skilled personnel [10].

Seroconversion to parasite antigens can occur as early as 1 month after infection [11]. Four common serological diagnostic methods for leishmaniasis include the direct agglutination test (DAT), indirect immunofluorescence assay (IFAT), enzyme-linked immunosorbent assay (ELISA) and immunochromatographic assay (ICA) [12–14]. The DAT has been the primary diagnostic tool in many developing countries due to its high sensitivity, specificity, simplicity and minimal equipment requirements [15–18]; however, it has gradually been replaced by other methods due to its time-consuming nature. The IFAT is recommended by the World Organization for Animal Health (WOAH) as a reference method []. However, the accuracy of this method depends on the skills and experience of trained personnel for interpreting the results under the microscope [19]. Although the ICA is suitable for on-site testing, its sensitivity is lower than that of the other methods [16]. ELISA is commonly used for serological testing, with clinical sample testing having a higher sensitivity than IFAT [20]. Nevertheless, the major challenges associated with the ELISA are skilled laboratory personnel, tedious washing and typically long incubation periods (approx. 2 h).

The homogeneous chemiluminescent immunoassay has been a widely used technology due to its fast “mix-and-measure” protocols without the need for repeated washing steps, thereby reducing both hands-on and total assay time, with high throughput and potential for automation [21]. This method relies on the interaction between donor microspheres and acceptor microspheres. A biological reaction brings the donor and acceptor microspheres into proximity (< 200 nm). Subsequently, under 680-nm laser irradiation, the photosensitizer on the donor microspheres converts oxygen in the surrounding environment into more active singlet oxygen that diffuses to the proximate acceptor microspheres, generating a series of chemiluminescent reactions with an emission wavelength of 615 nm [22].

In the study reported here, we not only used homogeneous chemiluminescence technology but also employed the dual-antigen sandwich assay format in immunological assays. Compared to the indirect immunological detection format using secondary antibodies, the dual-antigen sandwich assay of detecting antibodies offers two advantages. First, it involves specific recognition of the target antigen twice, thereby enhancing specificity on swine fever detection [23]. In addition, another study on swine fever reported that this method can detect all types of antibodies, particularly immunoglobulin M (IgM), which could be beneficial for early diagnosis [24].

Therefore, the objective of this study was to develop an accurate, rapid and user-friendly detection method for canine *Leishmania* antibody based on double-antigen sandwich homogeneous chemical luminescence.

## Methods

### Sample collection and categorization

A total of 149 canine serum samples were collected, of which 41 were confirmed by the China Agricultural University Veterinary Teaching Hospital to be positive for *Leishmania* based on the microscopic examination of lymph node aspirates. Also, 30 of the canine serum samples were confirmed to be *Leishmania* serological negative, including four samples from dogs infected with *Toxoplasma gondii*, three from dogs infected with *Shigella dysenteriae*, two from dogs infected with *Ehrlichia canis*, three from dogs infected with *Salmonella Typhi,* three from dogs infected with *Bacillus cereus*, two from dogs infected with *Giardia trichomonas*, two from Babesia-infected dogs and one from a *Tritrichomonas foetus*-infected dog. A total of 78 samples with unknown clinical information were collected from dogs in the endemic area of CanL in Beijing; clinical diagnostic information was lacking for these samples.

### Preparation and identification of diagnostic antigens

For serological testing of VL, the WOAH recommends the K39 diagnostic antigen, although the K9 and K26 antigens also show good diagnostic performance [25]. A recombinant protein with full-length K9, K26 repeat sequences and a single copy of K39 was designed as the diagnostic antigen, based on previous results [26, 27]. Nucleotide sequences of the three proteins were obtained from the National Center for Biotechnology Information (NCBI) database. Subsequently, the nucleotide sequences of the recombinant proteins were codon-optimized and His tags were added at the C-terminus. The GST tag was PCR-cloned into the pET-28a(+) vector at the* Nco*I-*Xho*I restriction sites. The target nucleotide sequence was synthesized, then subcloned into pET-28a(+) with seamless cloning using* Xho*I restriction sites. Nucleotide sequence synthesis and protein expression experiments were outsourced to Gene Universal (Anhui, China).

### Establishment of a homogeneous chemiluminescent dual-antigen sandwich assay for canine *Leishmania* antibodies

**Fig. 1:**
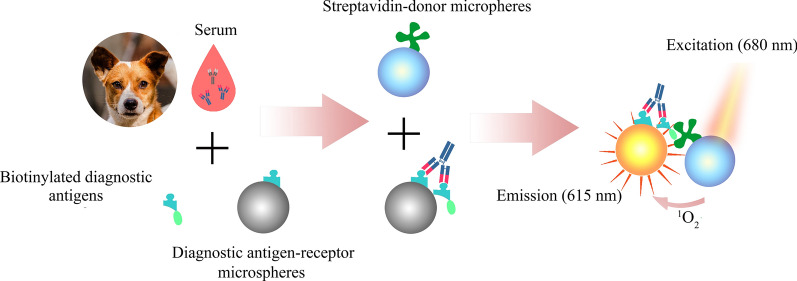
Schematic diagram of the reaction process

A clinical diagnostic method for detecting canine anti-*Leishmania* antibodies was established based on homogeneous chemiluminescent technology. First, diagnostic antigen-receptor microspheres, biotinylated diagnostic antigens and the test serum were sequentially added to a microplate. The mixture was incubated at 37 °C in the dark for 10 min, then streptavidin-modified donor microspheres were added, and incubation continued under the same conditions. In the presence of target antibodies, these components formed a multi-complex and generated an optical signal [28]. The reaction process is depicted in Fig. 1.

### Coupling diagnostic antigens to receptor microspheres

Diagnostic antigens were coupled to receptor microspheres using the carbodiimide method, following the manufacturer's recommendations (VDO Biotech, Suzhou Industrial Park, China). Initially, 100 μl of receptor microspheres (10 mg/ml) were pre-treated by sonication in 900 μl of MES (2-morpholinoethanesulphonic acid, 50 mM, pH 7.0). Subsequently, 20 mg of NHS (N-hydroxysuccinimide) and 20 mg of EDC (1-(3-dimethylaminopropyl)-3-ethylcarbodiimide hydrochloride) were dissolved in 1 ml of MES buffer; these solutions were freshly prepared as needed. NHS (10 μl) solution and EDC (5 μl) solution were then added to the cleaned receptor microspheres, and the resulting mixture was immediately mixed, then incubated at room temperature for 20 min to activate the receptor microspheres. After activation, receptor microspheres were centrifuged at 12,560 *g* for 20 min, then resuspended in 1000 μl of MES buffer and the centrifugation step repeated. Various amounts (0.0125, 0.025, 0.05, 0.1 and 0.2 mg) of diagnostic antigen were added to separate centrifuge tubes, with each tube containing 1 ml of activated receptor microsphere solution. The mixture was incubated at room temperature for 2 h. After labeling of the receptor microspheres with the diagnostic antigen, 100 μl of blocking solution (containing 20% bovine serum albumin [BSA] in 100 mM ethanolamine solution) was added, and incubation continued at room temperature for 1 h. Following completion of the blocking step, receptor microspheres were centrifuged at 12,560 *g* for 20 min, resuspended in 1000 μl of dilution solution (50 mM phosphate-buffered saline [PBS], pH 7.4) and the centrifugation step repeated. Labeled complexes were stored at 4 °C in the dark.

A checkerboard experiment was performed using receptor microspheres coupled with various concentrations of diagnostic antigens and a series of concentration gradients (0.03, 0.09, 0.27 and 0.81 μg/test) of biotinylated diagnostic antigens. Signal values and signal-to-noise ratios were plotted as a heatmap, and optimal conditions were selected.

### Optimization of diagnostic antigen-receptor microspheres and streptavidin-modified donor microspheres

Based on the determined concentration of biotinylated diagnostic antigen, concentrations of diagnostic antigen-receptor microspheres and streptavidin-modified donor microspheres were optimized. Concentration gradients were 0.125, 0.5, 1.0, 2.0, 4.0, and 8.0 μg/test for both components. Signal values were plotted as a stacked bar chart, and signal-to-noise ratios were depicted as a line graph, with the best conditions selected based on a combination of signal values and signal-to-noise ratios.

### Determination of reaction solution composition

The composition and concentrations of the blocking protein and surfactants in the reaction solution were optimized. Experiments were conducted using 0.1% casein, 2% BSA, 2% skim milk and 50% laboratory-standard blocking solution as blocking proteins. Various surfactants, including Tween-20, Triton X-100, polyvinyl pyrrolidone (PVP), and Tetronic 1307 (S9) surfactant, were tested. Signal values were depicted as a stacked bar chart, and signal-to-noise ratios displayed as a line graph, with best conditions selected based on a combination of signal values and signal-to-noise ratios.

### Optimization of reaction time

The reaction was divided into two steps. In the first step, diagnostic antigen-receptor microspheres, biotinylated diagnostic antigen and test serum were added to the antibody-coated microplate sequentially. This mixture was incubated at 37 °C in the dark, with shaking for a specified duration. In the second step, streptavidin-modified donor microspheres were added, and incubation continued under the same conditions. The reaction times in both steps were 10, 20, 30 and 40 min. Signal values were plotted as a stacked bar chart, and signal-to-noise ratios were depicted as a line graph, with optimal conditions chosen based on signal values plus signal-to-noise ratios.

### Determination of the cut-off value

The cut-off value was determined using 41 confirmed positive samples from the animal hospital and 30 negative canine sera (10 healthy canine sera and 20 canine sera with similar clinical signs but confirmed as non-leishmaniasis). The receiver operating characteristic (ROC) curves were plotted based on the detection signal values, and Youden's index was calculated to establish the optimal cut-off value.

Based on the determined cut-off value, five samples with positive signals ranging from strong to weak in strength were selected from confirmed positive samples and subsequently subjected to repeat experiments to calculate the intra-and inter-assay coefficients of variation.

### Method evaluation

A large-volume sample from the 41 confirmed positive samples that tested positive in both parasitological and immunofluorescence tests was selected as the positive quality control sample. This positive quality control sample was subjected to multiple dilutions for testing to determine analytical sensitivity; this was evaluated by testing 71 confirmed cases from the animal hospital.

As part of the evaluation process, the IDvet indirect ELISA test kit (Innovative Diagnostics, Grabels, France) was used as the reference [29]. Both the developed method and the indirect ELISA test kit were used to test 41 *Leishmania*-positive samples with known clinical diagnosis information, 30 negative samples and 78 samples for which there was no clinical information. Diagnostic sensitivity and specificity of the developed method were assessed based on these results. The consistency of the two methods was calculated using the SPSS calculation κ index.

## Results

### Identification of diagnostic antigens

**Fig. 2:**
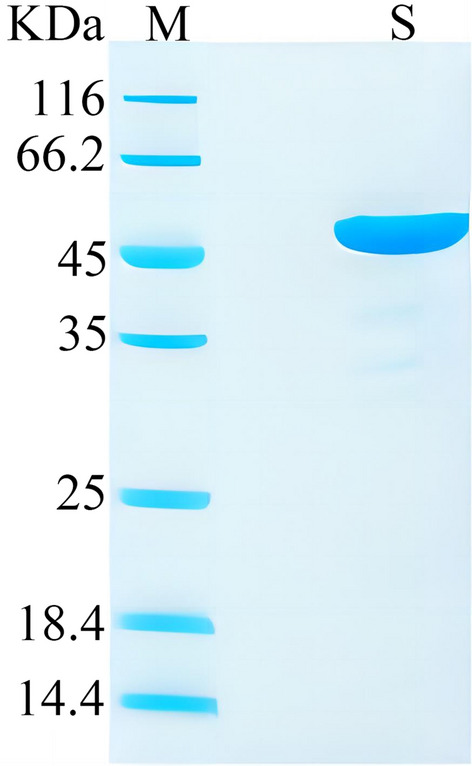
Sodium dodecyl sulfate-polyacrylamide gel electrophoresis of the prokaryotic expression product. The molecular weight of the obtained protein was approximately 45 kDa, as expected. Lanes:* M* marker,* S sample*

Sodium dodecyl sulfate-polyacrylamide gel electrophoresis (SDS-PAGE) was used to confirm the molecular weight of the diagnostic antigens obtained from prokaryotic expression. Following SDS-PAGE, staining revealed a single, clear band, and the observed protein size matched the expected molecular weight (45 kDa) (Fig. 2).

### Coupling of diagnostic antigens and receptor microspheres

**Fig. 3:**
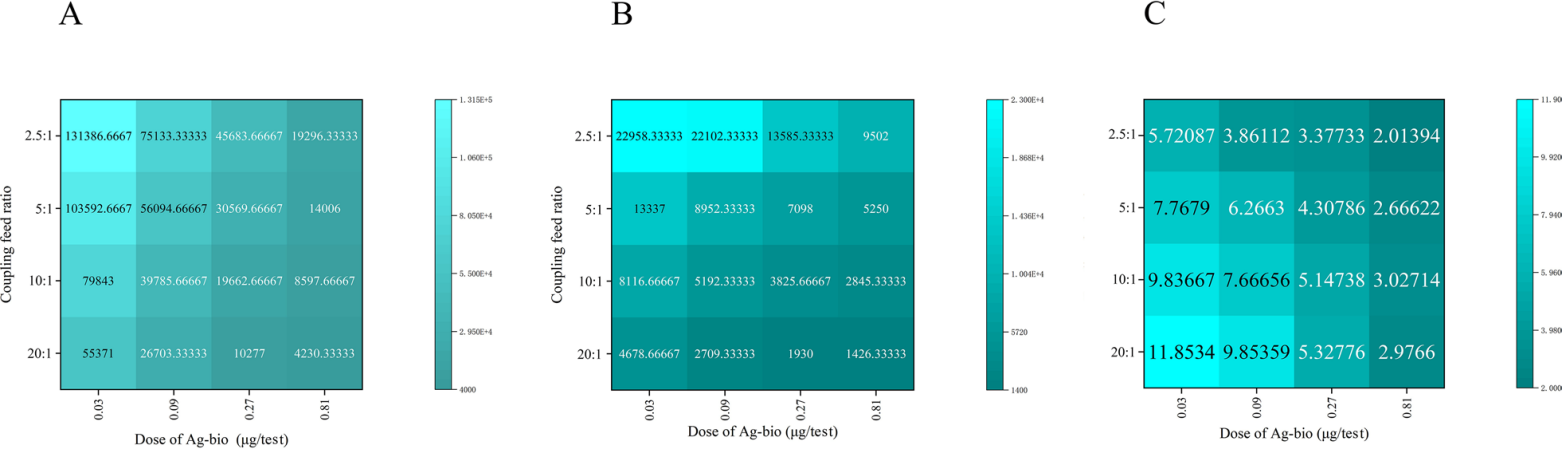
Checkerboard method was used to determine the optimal conditions for the coupling ratio of antigen protein to luminescent microspheres and for the quantity of biotinylated antigen protein.** A**–**C** Heat maps displaying positive signal values (**A**), negative signal values (**B**) and signal-to-noise ratio (**C**)

A checkerboard experiment was conducted by coupling diagnostic antigen-receptor microspheres with various concentrations of biotinylated diagnostic antigen. Positive and negative signal values, and signal-to-noise ratios of the test results were plotted as a heatmap (Fig. 3). As shown in Fig. 3, both signal values and signal-to-noise ratios decreased with increasing concentration of biotinylated diagnostic antigen. In terms of the coupling ratio, maintaining the microsphere quantity constant while increasing the amount of biotinylated diagnostic antigen increased the signal, but the signal-to-noise ratio showed the opposite trend. Based on these results, we determined the optimal coupling ratio of receptor microspheres to diagnostic antigen to be 20:1 (in terms of mass) and the optimal amount of biotinylated diagnostic antigen was set at 0.01 μg per test.

### Optimization of diagnostic antigen-receptor microspheres and streptavidin-modified donor microspheres

**Fig. 4:**
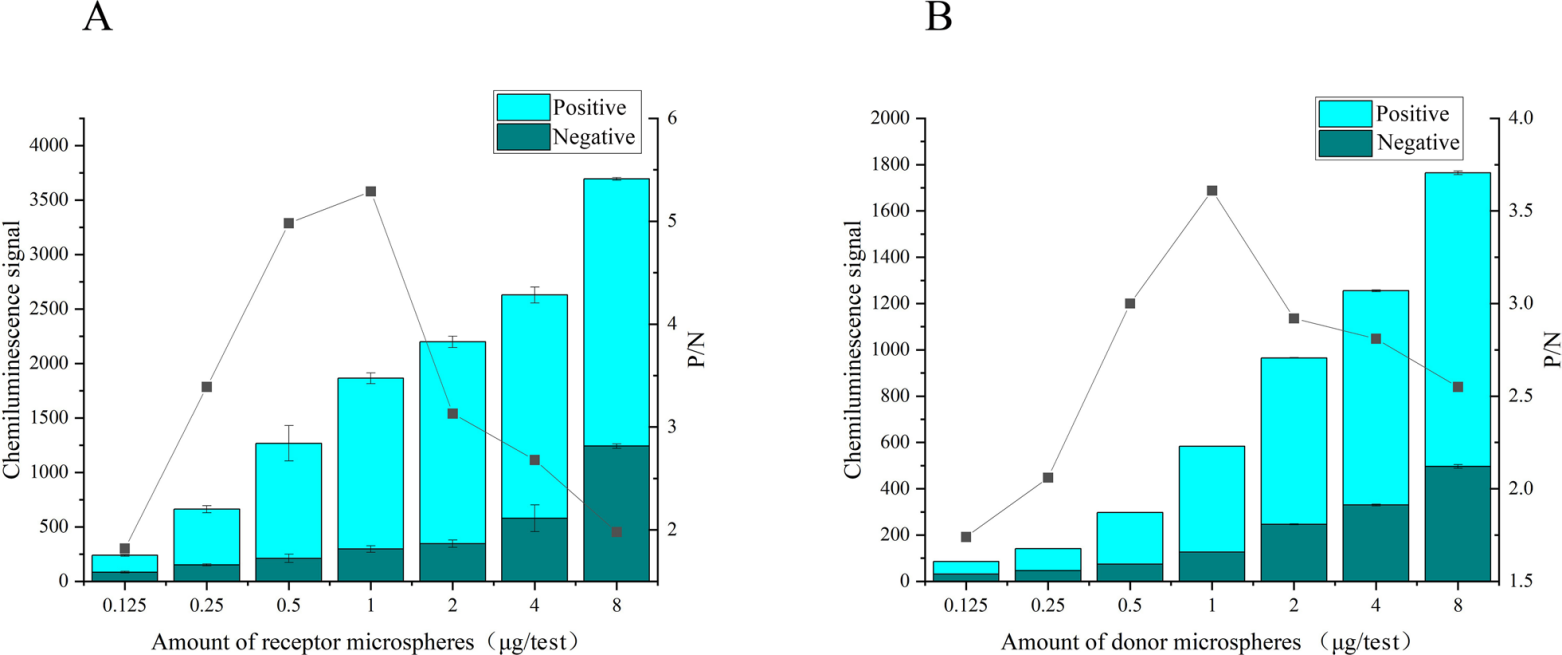
Optimization of the dosage of each reaction component.** A**,** B** Dosage of donor microspheres (**A**) and acceptor microspheres (**B**)

The quantities of the main components involved in the antigen–antibody reaction were optimized. In this experiment, the signal values were depicted as stacked bar graphs, while the signal-to-noise ratios were plotted as line graphs (Fig. 4). The signal values were found to consistently increase with increasing quantity, but the trend in the signal-to-noise ratios showed an initial rise followed by a subsequent decline. By integrating the performance of signal values and signal-to-noise ratios, the optimal conditions were determined following a balanced assessment. The optimal reaction quantities for the two components were established as 1.0 μg per assay.

### Determination of reaction solution system

**Fig. 5:**
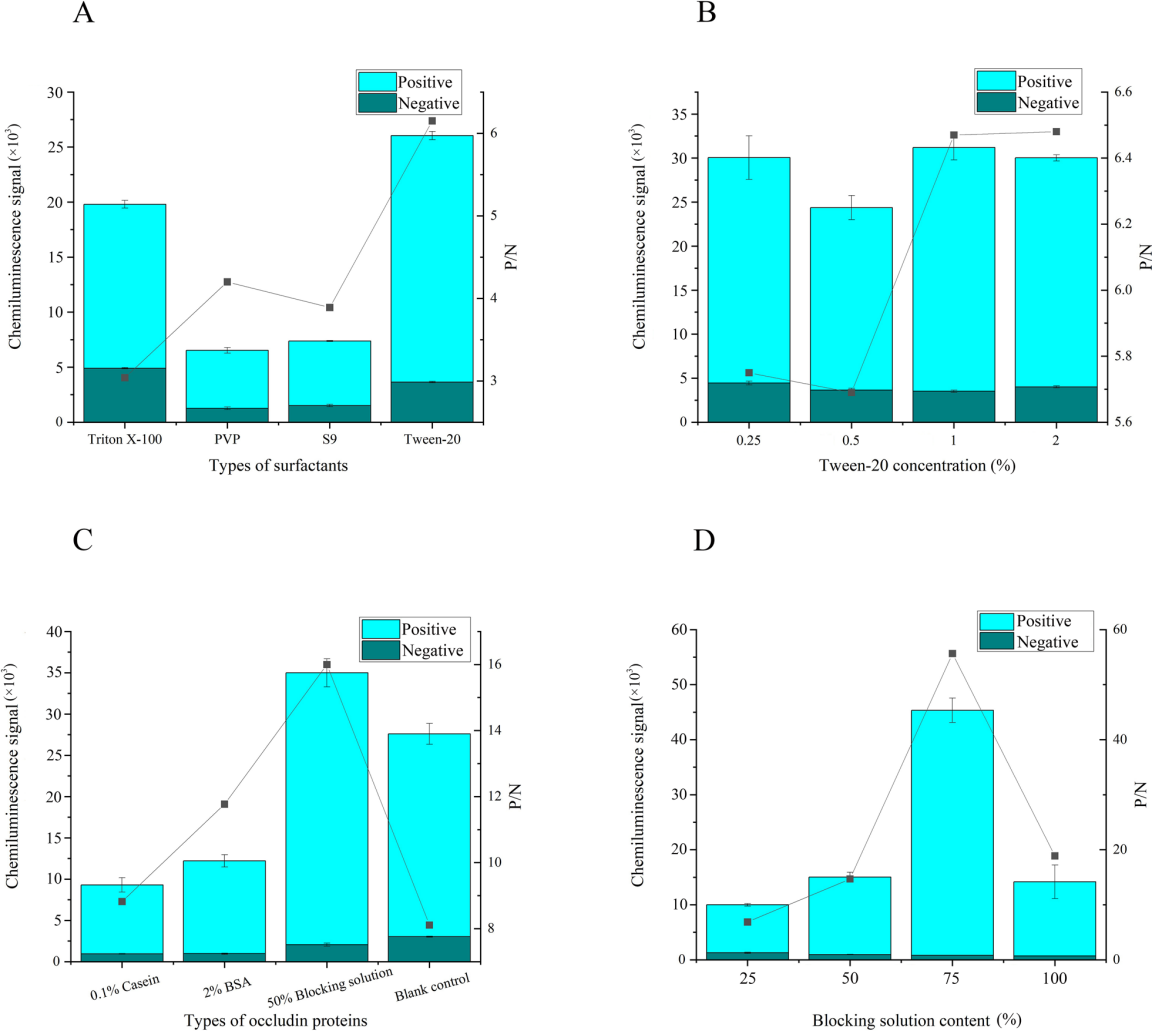
Determination of reaction buffer components. **A** Surfactant selection, **B** optimization of Tween-20 concentration, **C** selection of blocking protein types, **D** optimization of blocking solution concentration.* BSA* Bovine serum albumin,* PVP* polyvinyl pyrrolidone,* S9* Tetronic 1307 surfactant

Various proteins were utilized as blocking agents in the experiments, including 0.1% casein, 2% BSA, 2% skim milk and 50% laboratory blocking solution. We also tested different types of surfactants, including Triton X-100, PVP, S9 and Tween-20. As shown in Fig. 5, signal values were depicted as stacked bar graphs, and signal-to-noise ratios were illustrated as line graphs, with luminescent values represented on the left axis, signal-to-noise ratios displayed on the right axis and optimization parameters delineated on the abscissa. Following initial positive outcomes, further investigations were conducted on the laboratory blocking solution and Tween-20 to optimize the concentrations. As the concentration of the blocking solution increased, both the signal values and signal-to-noise ratios exhibited a similar trend, peaking at 75%. With increasing quantities of Tween-20, notable variations in the signal-to-noise ratios were observed, which stabilized after reaching 1%. The optimal concentrations for the blocking solution and Tween-20 were determined to be 75% and 1.0%, respectively.

### Optimization of reaction time

**Fig. 6:**
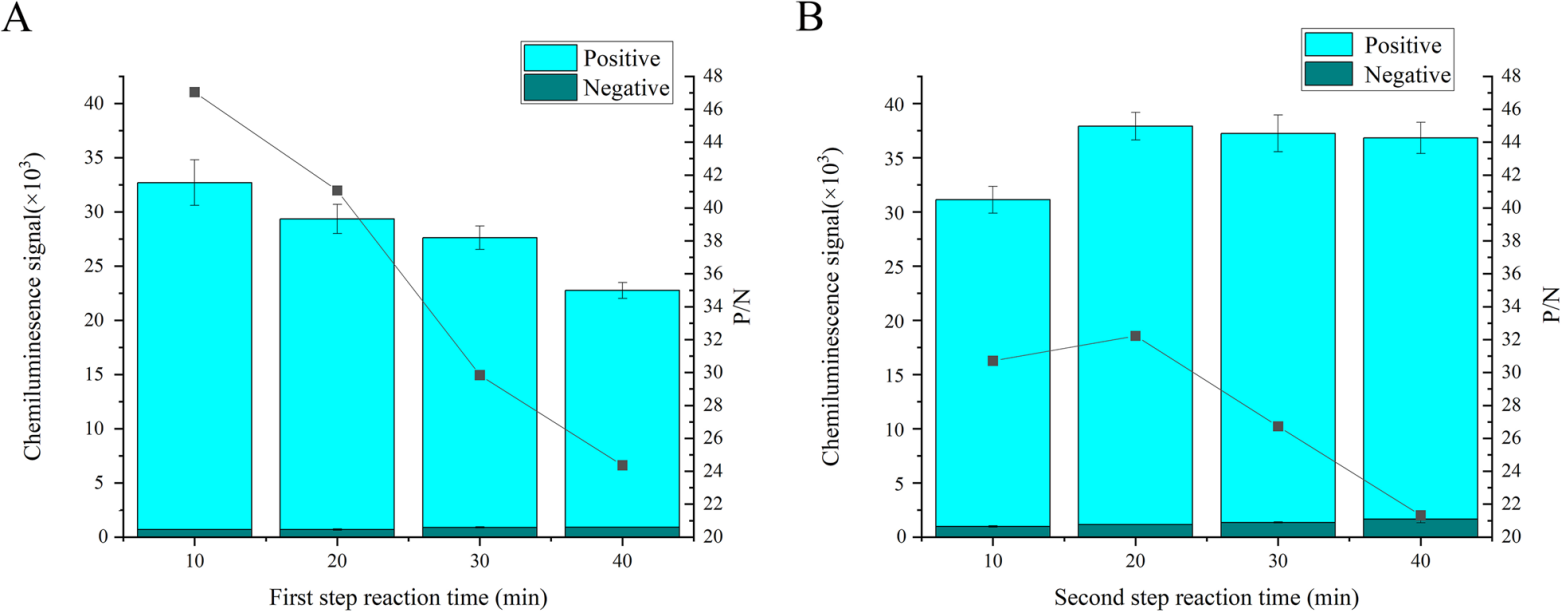
Optimization of reaction time. **A** Signal value and signal-to-noise ratio of the first step of the reaction. **B** Signal value and signal-to-noise ratio of the second step of the reaction

For the first step (reaction of diagnostic antigen-receptor microspheres, biotinylated diagnostic antigen and test serum) and the second step (reaction after the addition of streptavidin-modified donor microspheres), reaction times were set at 10, 20, 30 and 40 min. As shown in Fig. 6, signal values were plotted as stacked bar charts, and signal-to-noise ratios were depicted as a line graph. The figure shows that in the first step both signal values and signal-to-noise ratios exhibited a declining trend with increasing reaction time. The optimal signal-to-noise ratio was achieved at a reaction time of 20 min in the second step. The performance of signal value and signal-to-noise ratio were integrated to determine the optimal conditions after balanced evaluation, and the optimal reaction times for the first and second steps were determined to be 10 and 20 min, respectively.

### Determination of the threshold value

**Fig. 7:**
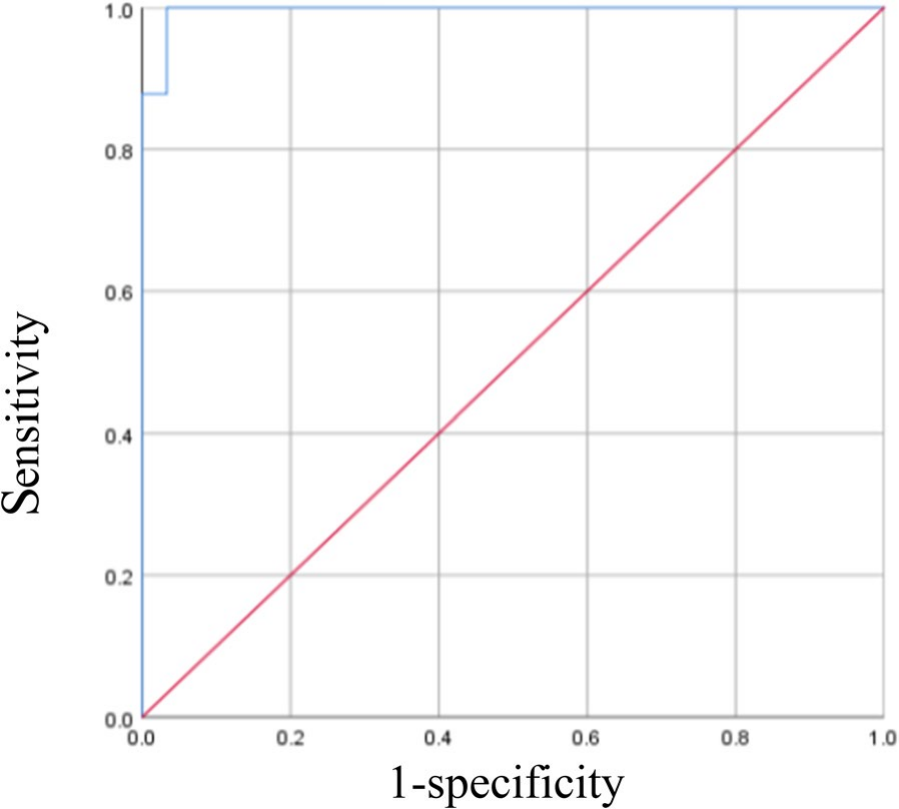
Receiver operating characteristic curve (ROC). Selection of the critical value through the ROC curve and combined sensitivity and specificity

For this study, 41 canine sera samples that had been confirmed to be *Leishmania*-positive through parasitological examination were used as positive detection data, and 30 canine serum samples determined to be *Leishmania*-negative by serological testing were used as negative data. We employed our newly established method to detect confirmed samples. Combining both sets of data, an ROC curve was plotted (Fig. 7), and the Youden index was calculated to determine the optimal threshold value (3150 Augmentation Units [AU]).

### Method evaluation

**Fig. 8:**
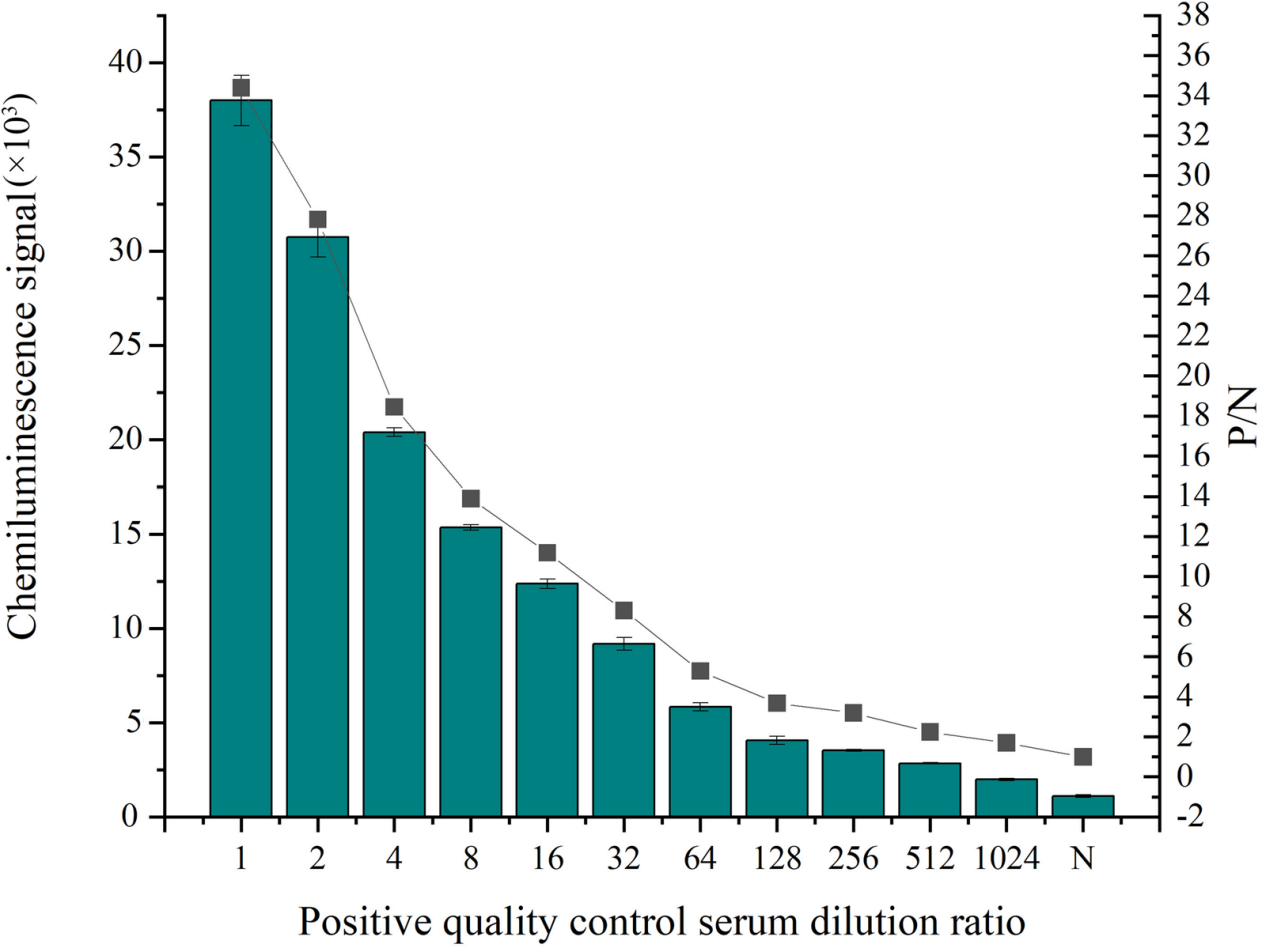
Analysis of sensitivity. The column diagram is the signal value, and the folding diagram is the signal-to-noise ratio

To determine the analytical sensitivity of the newly developed method, we diluted positive quality control samples to produce decreasing concentrations. We found that samples diluted by 256-fold tested positive for *Leishmania* (Fig. 8).

The analytical specificity was determined by testing 71 samples from the animal hospital, resulting in 100% specificity.

To determine diagnostic sensitivity, we tested 71 samples from the animal hospital. Diagnostic sensitivity of the method reported in this study and of the reference method was 100% (95% confidence interval [CI] 95.3–99.8%) and 95.1% (95% CI 89.5–98.1%), respectively.

For diagnostic specificity, we tested 71 samples from the animal hospital using both our newly developed method and the reference method. The diagnostic specificity of both methods was 93.3% (95% CI 90.0–96.5%).

In the consistency analysis, the detection rate for positive samples from the area where CanL is endemic, as determined by our newly developed method and the reference method was 32.1% (25/78) and 34.6% (27/78), respectively. Excluding samples considered to be "suspect" by the reference method, the remaining 76 clinical samples and 71 confirmed samples were used to determine consistency between the two methods. The Kappa value was 0.82 (95% CI 75.1–87.3%) (*P* < 0.01), indicating almost perfect agreement between our newly developed method and an indirect ELISA kit (Table 1).

**Table 1:** Cross-tabulation between this study and the IDvet ELISA test kit for detection of anti-*Leishmania* antibodies in canine serum

Test	Positivity/negativity	Current study		
		Anti-*Leishmania* antibodies positive	Anti-*Leishmania* antibodies negative	Total
ID Vet	Positive	65	0	65
	Negative	1	81	82
Total		66	81	147

## Discussion

The prevalence of canine leishmaniasis has increased worldwide in recent decades [33–35]. Serological diagnosis of this disease has primarily relied on the ELISA and ICA, but the disadvantages of these two methods limit their practical application. Although the ELISA has reasonable sensitivity, it is complex and time-consuming [30], and the ICA, which is known to be simple to execute and provide rapid testing, has relatively low sensitivity [16]. With advancements in technology, non-wash and highly sensitive homogeneous chemiluminescence techniques have been increasingly integrated into immunodiagnosis [21]. The sandwich assay method, which utilizes dual antigen detection for antibodies, was widely adopted due to its high specificity [24, 32]. Therefore, the aim of this study was to develop an accurate, rapid and user-friendly detection method for canine *Leishmania* antibodies based on double-antigen sandwich homogeneous chemiluminescence.

Various forms of diagnostic antigens have been used in the serological testing of canine leishmaniasis. The WOAH recommends the use of purified K39 protein as a diagnostic antigen []. A previous study indicated that a recombinant protein containing the full sequences of K26, K9 and K39 exhibited excellent diagnostic performance compared to K39 alone [29]. Additionally, another study confirmed the effectiveness of using a single-copy repeat sequence of K39, although its diagnostic performance was inferior to that of the full-length sequence [31]. However, research on the diagnostic efficacy of fusion proteins containing single-copy sequences of K9, K26 and K39 is scarce. One study demonstrated that fusion proteins containing partial copies of the K9 sequence, as well as the repeat sequences of K26 and K39, existed in dimeric form [27]. In this study, the GST tag was retained to maintain the monomeric form of the recombinant protein. The fusion proteins containing single-copy sequences of K9, K26 and K39 were successfully expressed, which is the minimum protein that is currently known to diagnose the *Leishmania* antibodies, demonstrating their effectiveness in detection.

Homogeneous chemiluminescence is a novel technology known for its high sensitivity and specificity [34]. In a previous study, the positive fluorescence signal for antibody detection typically reached 25,000 [21]. In our research, the positive signal value reached 40,000, whereas the negative signal value was < 3000. Therefore, signal expression for our method was within a reasonable range, and the distinction between positive and negative signals was highly significant.

The sensitivity and specificity of our newly developed method were 100% (41/41) and 97.7% (29/30), respectively. In comparison, the sensitivity and specificity of the indirect ELISA kit were 95.3% (39/42) and 97.67% (29/30), respectively, consistent with previously reported values [25]. In addition, our method had better sensitivity compared to other ELISA detection methods, which had sensitivities of 96% and 96.8% [26, 29]. Also, our method significantly reduced the amount of time and labor required, compared to ELISA. Additionally, compared to ICA, the sensitivity of our method dramatically increased to 100%, compared to 78% for ICA [15], with only with an additional 20 min [16].

In the field of on-site detection, the ICA and DAT are the major serological tests implemented. The DAT is hindered by the necessity for serum gradient dilution and extended reaction times (over 2 h) [12–15]. The major drawback of the ICA lies in its limited sensitivity [17]. The method used in our study facilitates the simultaneous testing of multiple samples, with high sensitivity and specificity, and the process from sample addition to result reading can be completed within 35 min. Nevertheless, the method in our study still necessitates a separate reading instrument. Additionally, although the laboratory skills required to perform our method are relatively straightforward compared to those needed for the ICA, our newly developed method still demands a certain level of operational proficiency.

## Conclusions

A novel double-antigen sandwich homogeneous chemical luminescence method to detect canine *Leishmania* antibodies was established, with high sensitivity and specificity, short incubation time and a simple protocol. This streamlined approach not only offers a sensitive and efficient method for clinical diagnosis but also has great potential for use in automated testing.

## Data Availability

The datasets supporting the conclusions of this article are included within the article.
